# Verbal memory formation across PET-based Braak stages of tau accumulation in Alzheimer’s disease

**DOI:** 10.1093/braincomms/fcad146

**Published:** 2023-05-18

**Authors:** Jaime Fernández Arias, Joseph Therriault, Emilie Thomas, Firoza Z Lussier, Gleb Bezgin, Cécile Tissot, Stijn Servaes, Sulantha S Mathotaarachchi, Dorothée Schoemaker, Jenna Stevenson, Nesrine Rahmouni, Min Su Kang, Vanessa Pallen, Nina Margherita Poltronetti, Yi-Ting Wang, Peter Kunach, Mira Chamoun, Kely M Quispialaya S, Paolo Vitali, Gassan Massarweh, Serge Gauthier, Maria N Rajah, Tharick Pascoal, Pedro Rosa-Neto

**Affiliations:** Faculty of Medicine, McGill University, Montreal, QC H3G 2M1, Canada; Department of Neurology and Neurosurger, McGill University Research Centre for Studies in Aging, Verdun, QC H4H 1R3, Canada; Faculty of Medicine, McGill University, Montreal, QC H3G 2M1, Canada; Department of Neurology and Neurosurger, McGill University Research Centre for Studies in Aging, Verdun, QC H4H 1R3, Canada; Faculty of Medicine, McGill University, Montreal, QC H3G 2M1, Canada; Department of Neurology and Neurosurger, McGill University Research Centre for Studies in Aging, Verdun, QC H4H 1R3, Canada; Department of Psychiatry, University of Pittsburgh, Pittsburgh, PA 15260, USA; Faculty of Medicine, McGill University, Montreal, QC H3G 2M1, Canada; Department of Neurology and Neurosurger, McGill University Research Centre for Studies in Aging, Verdun, QC H4H 1R3, Canada; Faculty of Medicine, McGill University, Montreal, QC H3G 2M1, Canada; Department of Neurology and Neurosurger, McGill University Research Centre for Studies in Aging, Verdun, QC H4H 1R3, Canada; Department of Psychiatry, University of Pittsburgh, Pittsburgh, PA 15260, USA; Faculty of Medicine, McGill University, Montreal, QC H3G 2M1, Canada; Department of Neurology and Neurosurger, McGill University Research Centre for Studies in Aging, Verdun, QC H4H 1R3, Canada; Faculty of Medicine, McGill University, Montreal, QC H3G 2M1, Canada; Department of Neurology and Neurosurger, McGill University Research Centre for Studies in Aging, Verdun, QC H4H 1R3, Canada; Department of Psychiatry, Massachusetts General Hospital, Boston, MA 02114, USA; Faculty of Medicine, McGill University, Montreal, QC H3G 2M1, Canada; Department of Neurology and Neurosurger, McGill University Research Centre for Studies in Aging, Verdun, QC H4H 1R3, Canada; Faculty of Medicine, McGill University, Montreal, QC H3G 2M1, Canada; Department of Neurology and Neurosurger, McGill University Research Centre for Studies in Aging, Verdun, QC H4H 1R3, Canada; Faculty of Medicine, McGill University, Montreal, QC H3G 2M1, Canada; Department of Neurology and Neurosurger, McGill University Research Centre for Studies in Aging, Verdun, QC H4H 1R3, Canada; Faculty of Medicine, McGill University, Montreal, QC H3G 2M1, Canada; Department of Neurology and Neurosurger, McGill University Research Centre for Studies in Aging, Verdun, QC H4H 1R3, Canada; Faculty of Medicine, McGill University, Montreal, QC H3G 2M1, Canada; Department of Neurology and Neurosurger, McGill University Research Centre for Studies in Aging, Verdun, QC H4H 1R3, Canada; Faculty of Medicine, McGill University, Montreal, QC H3G 2M1, Canada; Department of Neurology and Neurosurger, McGill University Research Centre for Studies in Aging, Verdun, QC H4H 1R3, Canada; Faculty of Medicine, McGill University, Montreal, QC H3G 2M1, Canada; Department of Neurology and Neurosurger, McGill University Research Centre for Studies in Aging, Verdun, QC H4H 1R3, Canada; Faculty of Medicine, McGill University, Montreal, QC H3G 2M1, Canada; Department of Neurology and Neurosurger, McGill University Research Centre for Studies in Aging, Verdun, QC H4H 1R3, Canada; Faculty of Medicine, McGill University, Montreal, QC H3G 2M1, Canada; Department of Neurology and Neurosurger, McGill University Research Centre for Studies in Aging, Verdun, QC H4H 1R3, Canada; Department of Neurology and Neurosurger, McGill University Research Centre for Studies in Aging, Verdun, QC H4H 1R3, Canada; Department of Radiochemistry, Montreal Neurological Institute, Montreal, QC H3A 2B4, Canada; Department of Neurology and Neurosurger, McGill University Research Centre for Studies in Aging, Verdun, QC H4H 1R3, Canada; Department of Psychiatry, Douglas Mental Health University Institute, Verdun, QC H4H 1R3, Canada; Department of Psychiatry, University of Pittsburgh, Pittsburgh, PA 15260, USA; Department of Psychiatry, Douglas Mental Health University Institute, Verdun, QC H4H 1R3, Canada; Department of Psychiatry, University of Pittsburgh, Pittsburgh, PA 15260, USA; Faculty of Medicine, McGill University, Montreal, QC H3G 2M1, Canada; Department of Neurology and Neurosurger, McGill University Research Centre for Studies in Aging, Verdun, QC H4H 1R3, Canada; Department of Neurology and Neurosurgery, Montreal Neurological Institute, Montreal, QC H3A 2B4, Canada

**Keywords:** delayed recall, recognition memory, PET-Braak staging, tau PET, Alzheimer’s disease

## Abstract

A classical early sign of typical Alzheimer’s disease is memory decline, which has been linked to the aggregation of tau in the medial temporal lobe. Verbal delayed free recall and recognition tests have consistently probed useful to detect early memory decline, and there is substantial debate on how performance, particularly in recognition tests, is differentially affected through health and disease in older adults. Using *in vivo* PET-Braak staging, we investigated delayed recall and recognition memory dysfunction across the Alzheimer’s disease spectrum. Our cross-sectional study included 144 cognitively unimpaired elderly, 39 amyloid-β+ individuals with mild cognitive impairment and 29 amyloid-β+ Alzheimer’s disease patients from the Translational Biomarkers in Aging and Dementia cohort, who underwent [^18^F]MK6240 tau and [^18^F]AZD4694 amyloid PET imaging, structural MRI and memory assessments. We applied non-parametric comparisons, correlation analyses, regression models and voxel-wise analyses. In comparison with PET-Braak Stage 0, we found that reduced, but not clinically significant, delayed recall starts at PET-Braak Stage II (adjusted *P* < 0.0015), and that recognition (adjusted *P* = 0.011) displayed a significant decline starting at PET-Braak Stage IV. While performance in both delayed recall and recognition related to tau in nearly the same cortical areas, further analyses showed that delayed recall rendered stronger associations in areas of early tau accumulation, whereas recognition displayed stronger correlations in mostly posterior neocortical regions. Our results support the notion that delayed recall and recognition deficits are predominantly associated with tau load in allocortical and neocortical areas, respectively. Overall, delayed recall seems to be more dependent on the integrity of anterior medial temporal lobe structures, while recognition appears to be more affected by tau accumulation in cortices beyond medial temporal regions.

## Teaser

Fernández-Arias *et al*. report early delayed recall deficits concomitant with tau accumulation in the anterior medial temporal lobe, and recognition dysfunction with further tau buildup in neocortical regions in the Alzheimer’s disease spectrum. The study underscores the advantage of using PET-Braak staging to characterize memory-related deficits in Alzheimer’s disease.

## Graphical Abstract

**Graphical abstract:**
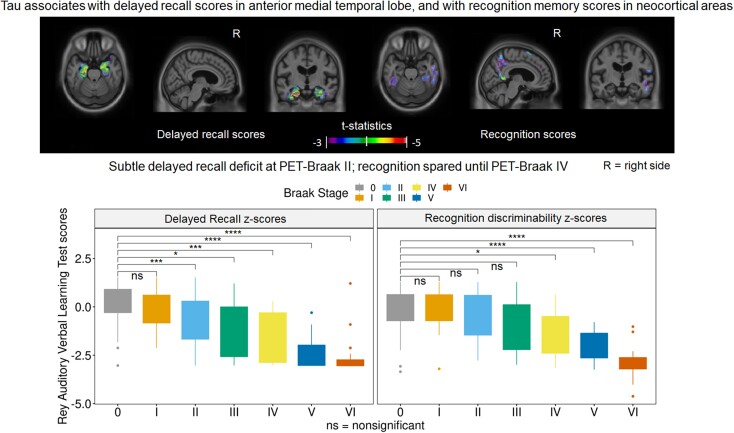

## Introduction

Alzheimer’s disease is characterized by neuropathological hallmarks that consist of the accumulation of extracellular amyloid-β plaques and intracellular neurofibrillary tangles,^1^ which are composed of insoluble fibrillary deposits of hyperphosphorylated tau.^2^ In the last two decades, advances in neuroimaging techniques for the *in vivo* assessment of amyloid-β and tau have enabled a move towards a biological definition of the disease.^3,4^ To this respect, cognitive testing that can be reliably linked to biomarkers may play a crucial role in staging disease. Recent studies have proposed Alzheimer’s disease staging using tau PET following a framework similar to that described by Braak and Braak.^5-9^ However, the cognitive characterization of such PET-Braak staging system remains to be characterized.

Decline in the formation of verbal memory is commonly observed in preclinical stages of typical Alzheimer’s disease,^10^ and it is often measured using list-learning tests, such as the California verbal learning test,^11^ the delayed recall portion of the Alzheimer’s disease assessment scale-cognitive subscale,^12^ or the Rey auditory verbal learning test (RAVLT).^13^ Neuropsychological and brain imaging studies have consistently implicated medial temporal lobe (MTL) structures in verbal memory construction difficulties,^14-16^ and lesions to this region result in profound amnesia.^17-19^ Indeed, these deficits have been associated with the accumulation of tau pathology precisely in MTL areas in Alzheimer’s disease.^20,21^ However, not all aspects of memory are interchangeable. A distinction can be made between delayed free recall and recognition memory. Verbal free recall tasks typically involve a list of words that is repeatedly presented over a short period of time (learning phase), and a delayed free recall test 20–25 min later. Subsequently, a verbal recognition test requires participants to say whether or not (yes/no; old/new) a word has been shown at the learning phase. Prior neuropsychological and neuroimaging studies indicate that MTL, prefrontal and inferior parietal regions may be implicated in free recall and, overall, memories that rely on retrieval of contextual information, and highlight these tasks’ demand on mnemonic, strategic organization and cognitive control processes in aging and dementia.^22-28^ Verbal item recognition has been associated with damage to anterior portions of the MTL, including perirhinal (PRC) and entorhinal cortices.^29-32^ The degree to which these different aspects of memory are affected in cognitively unimpaired (CU) older adults, prodromal Alzheimer’s disease and clinical Alzheimer’s disease is highly contested.^33^ Although difficulties in interpretation arise due to differences in study design, the manner in which memory testing was carried out, or participants’ inclusion criteria across studies, generally speaking, there is little doubt that delayed free recall is affected in CU older adults, and that further deterioration occurs in preclinical and, particularly, clinical Alzheimer’s disease. However, the sequence of decline for recognition memory remains to be elucidated.

As tau deposits in all areas mentioned above, our goal was to assess how delayed recall and recognition evolve throughout the natural history of Alzheimer’s disease from a PET-Braak perspective and to inspect brain areas where vulnerability to tau pathology adversely affects different aspects of auditory verbal memory. Since decreases in delayed recall seem certain in CU elderly individuals, while deficits in recognition are disputed even in preclinical Alzheimer’s disease, and delayed recall performance has been found to associate with tau load in anterior MTL of CU elderly, we hypothesized that delayed recall deficits would already be affected by the accumulation of tau in early PET-Braak stages, while recognition reductions would be observed at a later PET-Braak stage. For the same reasons, we predicted that delayed recall would be primarily associated with tau deposition in MTL regions, while recognition memory would relate to rather posterior neocortical brain areas.

## Materials and methods

### Participants

We assessed 144 CU older adults (>50 years old), 39 amyloid-β+ individuals with mild cognitive impairment (MCI) and 29 amyloid-β+ individuals with dementia due to Alzheimer’s disease recruited for the Translational Biomarkers of Aging and Dementia cohort^34^ who underwent [^18^F]AZD4694 scans for amyloid-β-PET, [^18^F]MK6240 scans for tau PET and MRI scans. They also completed comprehensive clinical, cognitive and neuropsychological assessments. CU participants had no objective cognitive impairment and a clinical dementia rating score of 0. Individuals with MCI had either subjective or objective cognitive impairment or both, a clinical dementia rating score of 0.5, and were able to carry on with activities of daily living. Alzheimer’s clinical syndrome patients were mildly or moderately demented, had a clinical dementia rating score between 0.5 and 2, and met the National Institute on Aging–Alzheimer’s Association criteria for probable Alzheimer’s disease according to a specialist.^35^ Exclusion criteria encompassed psychiatric, neurological or systemic conditions that were not controlled by medication, concurrent substance abuse, recent major surgery, recent head trauma and MRI/PET safety contraindications. The research project obtained approval from the Montreal Neurological Institute PET working committee and the Douglas Mental Health University Institute Research Ethics Board. Consent was acquired for all participants in written form.

### MRI and PET acquisition and processing

Structural MRI scans were performed at the Montreal Neurological Institute on a 3 T Siemens Magnetom scanner using a standard head coil. T1-weighted images were acquired for all participants. [^18^F]AZD4694 PET and [^18^F]MK6240 PET scans were conducted using a Siemens high-resolution research tomograph. [^18^F]MK6240 PET images were collected between 90 and 110 min following intravenous bolus injection of the radiotracer, and reconstruction was accomplished using an ordered subset expectation maximization algorithm on a 4D volume with four frames (4 × 300 s), as described elsewhere.^36^ The average [^18^F]MK6240 dose injected was 6.28 mCi (SD = 0.74). [^18^F]AZD4694 PET images were acquired 40–70 min after the intravenous bolus injection of the radiotracer. For reconstruction, we used the same ordered subset expectation–maximization algorithm on a 4D volume with three frames (3 × 600 s).^37^ The average [^18^F]AZD4694 PET dose was 6.45 mCi (SD = 0.61). At the end of each PET session, a 6-min transmission scan with a rotating ^137^Cs point source was performed for attenuation correction. Corrections for motion, decay, dead time and random and scattered coincidences were also applied. Linear registration to the T1-weighted image space was computed for PET images, and linear and non-linear registration to the Alzheimer’s disease neuroimaging initiative template was computed for T1-weighted images.^38^ Meninges were removed from [^18^F]MK6240 images in native space before transformations and blurring to attenuate the influence of meningeal off-target binding on adjacent brain regions.^39^ Further linear and non-linear registration of PET images to the Alzheimer’s disease neuroimaging initiative template was completed using the aforementioned transformations from the T1-weighted image to Alzheimer’s disease neuroimaging initiative space and from the PET image to the T1-weighted image space. standardized uptake value ratios (SUVRs) were calculated to estimate abnormal amounts of radiotracer binding across the brain with respect to a reference region where values are deemed stable across participants and conditions.^40^ This region was the inferior cerebellar grey matter for [^18^F]MK6240 SUVRs,^39,41^ as derived from the Spatially Unbiased Infratentorial Template cerebellum atlas.^42^ As for [^18^F]AZD4694 SUVRs, the whole cerebellum grey matter was designated as the reference region.^37^ SUVR values for both radiotracers were calculated using R software (3.6.3 version). Spatial smoothing of PET images was performed to achieve an 8-mm full-width at half-maximum resolution. A global [^18^F]AZD4694 SUVR composite was calculated using the following brain regions: precuneus, prefrontal, orbitofrontal, parietal, temporal and cingulate cortices.^37,43^ An [^18^F]AZD4694 SUVR > 1.55 indicated amyloid-β positivity, as previously established.^37^

### PET-Braak staging

A full description of the method for PET-based Braak staging can be found elsewhere.^39,44^ Braak stages were based on anatomical brain regions suggested by Braak.^45,46^ PET-Braak-defined stages included: transentorhinal cortex (PET-Braak I), entorhinal cortex and hippocampus (PET-Braak II), inferior temporal neocortex (PET-Braak III), association cortices (PET-Braak IV and V) and primary sensory cortices (PET-Braak VI).^6,39,44^ Participants were assigned PET-Braak stages based on the latest stage where tau PET abnormality was identified by an automatic pipeline.^39^ Segregation into higher PET-Braak stages was determined only if the cutoff in lower stages was also reached. The threshold for tau PET abnormality across PET-Braak regions was defined as 2.5 SD higher than the mean SUVR of CU young adults, a previously set standard.^36,39^ Consistent with the correspondence between the probability of Alzheimer’s disease dementia diagnosis and Braak stages,^47^ 76% of our PET-Braak I and II participants were CU elderly and only 6–7% of them had a diagnosis of probable Alzheimer’s disease. By contrast, 72% of participants assigned PET-Braak Stages V or VI had such diagnosis.

### Verbal memory assessment

Memory was assessed using delayed free recall and recognition measures of the RAVLT.^13^ Raw delayed recall scores were z-transformed using mean and SD values from the CU older adults. Recognition discriminability (d′) and bias (C′) scores were calculated using the signal detection theory paradigm^48^ where mean and SD values from CU older adults were also the reference. Recognition d′ scores reflect the difference between the curves of correctly identified items (hits) and incorrectly selected items (false alarms). The higher the score, the better participants are to distinguish previously presented items. C′ scores are complementary to d′ and indicate an inclination to guess (negative score) or not (positive score). No bias is given by a score of zero. We followed Stanislaw and Todorov’s^49^ formulas to calculate these scores using the NORMSINV package of LibreOffice Calc software, with corrections applied to hit and false alarm’s rates. This function returns the inverse of the standard normal cumulative distribution.

### Statistical methods

We used R version 3.6.3 for all analyses, with the exception of voxel-wise analyses. Demographic characteristics were evaluated using *t*-tests and *χ*^2^. We calculated mean images of [^18^F]MK6240 SUVR and [^18^F]AZD4694 SUVR according to the PET-based Braak stage using Brain Imaging Center Medical Imaging Network Common Data Form (BIC MINC) toolbox. Delayed recall and recognition scores were compared between PET-based Braak stages using Kruskal–Wallis H tests, since normality assumptions were not met and some PET-Braak stage groups had a low number of participants. Mann–Whitney U-tests were conducted with the false discovery rate correction for multiple comparisons. Associations between PET-Braak stage tau SUVR and memory scores were evaluated using Pearson correlations and linear regression models, where RAVLT bias C′ scores,^48^ [^18^F]AZD4694 SUVR, age, sex, apolipoprotein E (*APOE*) status and years of education were entered as covariates. Voxel-wise analyses were performed to visualize the relationship between tau PET and memory scores in the brain using VoxelStats.^50^ We included [^18^F]AZD4694 SUVR, age, sex, *APOE* status and years of education as covariates in two models. We added the remaining target memory scores (i.e. RAVLT d′ scores if delayed recall was the outcome) and RAVLT bias C′ scores, and replaced raw [^18^F]AZD4694 SUVR values with amyloid positivity in two other models.

## Results

Demographic and clinical information is summarized in Table 1. All variables in regression models had a variance inflation factor < 5 and a tolerance > 0.2, which are not considered to indicate problematic levels of multicollinearity.^51^

**Table 1:** Demographics^a^

	CU	MCI	*P-*value	Alzheimer’s disease	*P-*value
*N*	144	39		29	
Age, years, mean (SD)	71.7 (5.9)	72 (5)	0.69	66.9 (8)	0.005
Female, *n* (%)	95 (66)	26 (67)	0.94	17 (59)	0.45
Education, years, mean (SD)	15.4 (3.7)	15.7 (3.7)	0.59	14.8 (3.1)	0.38
APOE ε4 carriers (%)	36 (25)	22 (56)	<0.001	17 (59)	<0.001
[^18^F]AZD4694 SUVR mean (SD)	1.45 (0.34)	2.35 (0.48)	<0.001	2.51 (0.45)	<0.001

a *P*-values indicate values assessed with independent samples *t*-tests for each variable except sex and APOE ε4 status, where contingency *χ*^2^ tests were performed (corrected with the Bonferroni procedure for multiple comparisons; significant if *P* < 0.025). *P*-values reported are for comparisons with CU subjects: the leftmost *P*-values reflect comparisons between CU and MCI groups; the rightmost *P*-values reflect comparisons between CU and Alzheimer’s disease groups.

SUVR, standardized uptake value ratio; CU, cognitively unimpaired; MCI, mild cognitive impairment.

**Figure 1:**
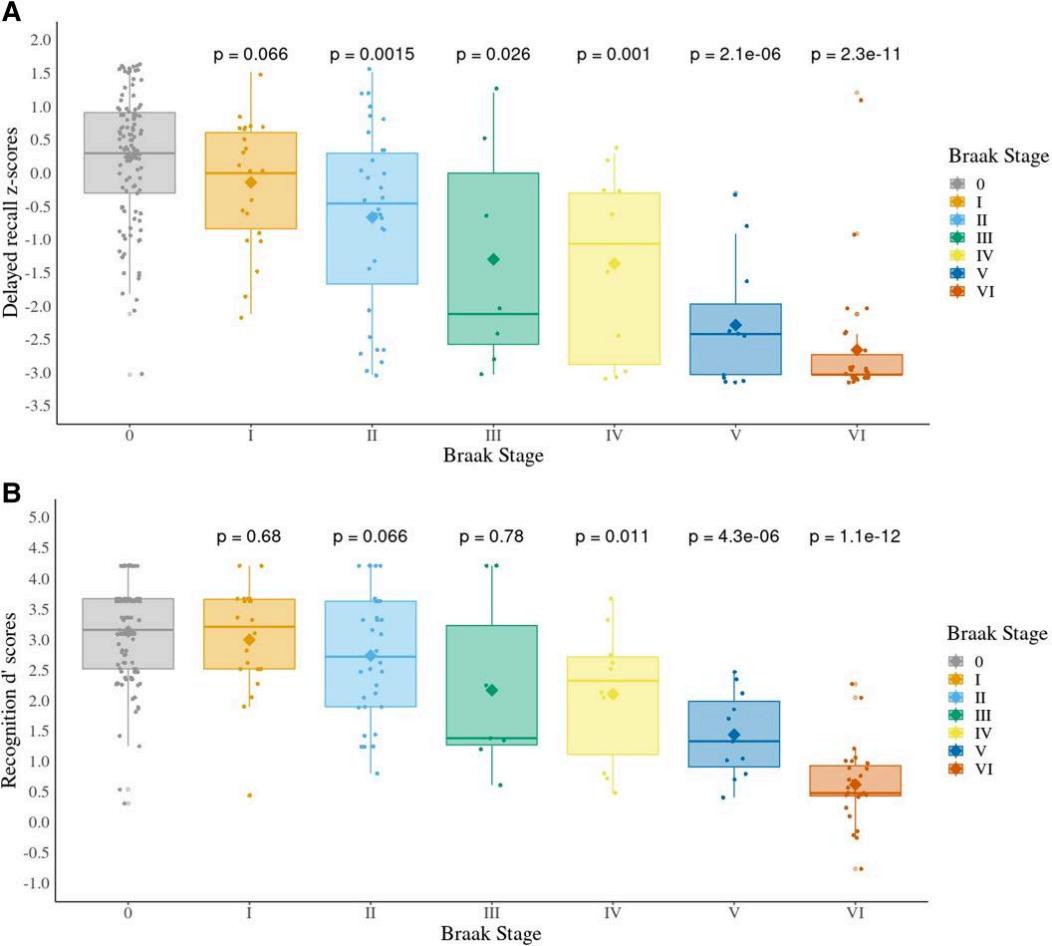
**While delayed recall declines at PET-Braak Stage II, recognition deficits are observed at PET-Braak Stage IV. Mean and median memory scores as assessed by *in vivo* tau PET.** (**A**) RAVLT delayed recall z-scores. (**B**) RAVLT-derived discriminability d′ (recognition) scores. Adjusted *P*-values are displayed for the comparison between individuals in Braak 0 and individuals in more advanced Braak stages. Kruskal–Wallis H tests were applied. For multiple comparisons, we used Mann–Whitney U-tests, with false discovery rate correction. 0: CU elderly with no significant tau; I–VI: PET-Braak stage. Scores from CU elderly were used to calculate z-scores.

We tested the hypothesis that memory impairment would be observed across the PET-Braak staging spectrum, with a stark decline around PET-Braak III or PET-Braak IV. Indeed, delayed recall memory was significantly affected across PET-Braak stages, *H*(6) = 82.2, *P* < 0.001, *ε*^2^=0.39. Six *post hoc* Mann–Whitney *U*-tests, corrected with the false discovery rate procedure, revealed that delayed recall z-scores of PET-Braak 0 participants were not significantly different from those of PET-Braak I (*W* = 1455, adjusted *P* = 0.066), but reached significance when compared to PET-Braak II (*W* = 2344, adjusted *P* = 0.002), PET-Braak III (*W* = 563, adjusted *P* = 0.026), PET-Braak IV (*W* = 882, adjusted *P* = 0.001), PET-Braak V (*W* = 1112, adjusted *P* < 0.001) and PET-Braak VI (*W* = 2424, adjusted *P* < 0.001) individuals (Fig. 1A).

Recognition memory was also significantly impaired across groups, *H*(6) = 80.8, *P* < 0.001, *ε*^2^=0.38. *Post hoc U* tests showed that, while d′ scores of PET-Braak 0 individuals did not significantly differ from d′ scores of PET-Braak I (*W* = 1230, adjusted *P* = 0.68), PET-Braak II (*W* = 2094, adjusted *P* = 0.066) and PET-Braak III (*W* = 526, adjusted *P* = 0.078) participants, they did significantly fluctuate when compared to those of PET-Braak IV (*W* = 812, adjusted *P* = 0.011), V (*W* = 1098, adjusted *P* < 0.001) and VI (*W* = 2496, adjusted *P* < 0.001) subjects (Fig. 1B).

We further tested the robustness of these results by collapsing participants into PET-Braak I–II, III–IV and V–VI groups (Supplementary Fig. 1). Our results were confirmed by these additional analyses.

In parallel, our analyses focused on examining the hypotheses that memory would be predicted by accumulation of tau in key memory-related areas of the brain beyond the influence of pathological amyloid-β and other covariates, and that this association would be noticeably stronger in MTL areas for delayed recall memory and in posterior neocortical areas for recognition memory.

**Figure 2:**
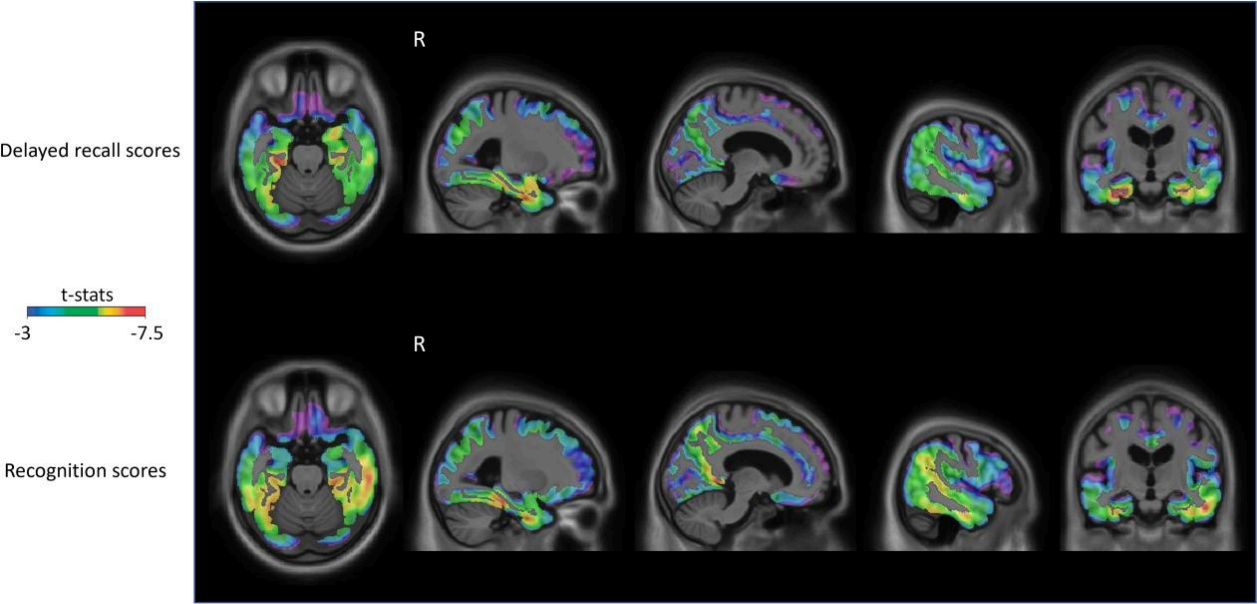
**PET scans revealed that delayed recall and recognition scores associate with tau in the same areas, but while delayed recall’s strongest associations with tau arise in antero-mesial and latero-temporal regions, recognition’s strongest associations with tau predominate in posterior temporal and parietal cortices.** T-statistical parametric maps were corrected for multiple comparisons using a random field theory cluster threshold of *P* < 0.001, overlaid on the Alzheimer's Disease Neuroimaging Initiative reference template. Two linear regression models were used, were either delayed recall z-scores or recognition d′ scores were entered as outcome variables. Age, sex, years of education, *APOE* status and amyloid-β SUVR were used as covariates.

In order to test these hypotheses, we first extracted SUVR values from areas associated with each of the PET-Braak stages and assessed their correlation with both delayed recall z-scores and recognition d′ scores. Tau accumulation in early PET-Braak areas strongly correlated with delayed recall scores, whereas this association was strongest in PET-Braak region I, and was similarly high for PET-Braak II, III and IV for recognition scores. These results are summarized in Table 2. Using the *cocor* package of R,^52^ and applying false discovery rate correction for multiple comparisons, we found significant differences for correlations between delayed recall scores and PET-Braak I versus II (*P* = 0.002), I versus III (*P* = 0.008), I versus IV, V or VI (*P* < 0.001 for all), II versus V (*P* = 0.003), II versus VI (*P* < 0.001), III versus V or VI (*P* < 0.001 for both), IV versus V (*P* < 0.001), and between IV versus VI (*P* = 0.004) SUVRs; as well as a marginal difference for PET-Braak II versus IV (*P* = 0.054) SUVRs. For recognition scores, correlations were significantly different between PET-Braak I versus II (*P* = 0.003), I versus V (*P* = 0.005), I versus VI (*P* < 0.001), II versus VI (*P* = 0.012), III versus V (*P* = 0.011), III versus VI (*P* < 0.001), IV versus V or VI (*P* < 0.001 for both) and V versus VI (*P* = 0.022) SUVRs. When comparing the correlations between PET-Braak stage SUVR and type of memory score, we found no significant differences. *P*-values were derived from Hittner *et al.*,’ (2003) method.^53^ Furthermore, we conducted voxel-wise analyses, which revealed that tau affects delayed recall and recognition memory scores effectively in virtually the same brain cortical areas (Fig. 2; random field theory corrected at *P* < 0.001). The strength of the relationships widely varied by memory type. While delayed recall showed the strongest associations with tau PET in anterior MTL, posterior hippocampus, parahippocampal cortex (PHC), lateral temporal lobe, and fusiform cortex, recognition memory exhibited the strongest associations with tau burden in the lateral temporal lobe, fusiform cortex, posterior cingulate cortex/precuneus, temporoparietal and parietal areas. These results were independent of amyloid-β, age, sex, years of education and *APOE* status.

**Table 2:** Correlation coefficients between Braak stage tau SUVR and memory scores^a^

	DR z-scores	*P-*value	d′ scores	*P-*value
*N*	212		212	
Braak Stage I SUVR	−0.68 (−0.75 to −0.60)	<0.001	−0.66 (−0.73 to −0.57)	<0.001
Braak Stage II SUVR	−0.63 (−0.71 to −0.54)	<0.001	−0.61 (−0.69 to −0.52)	<0.001
Braak Stage III SUVR	−0.59 (−0.67 to −0.49)	<0.001	−0.61 (−0.69 to −0.52)	<0.001
Braak Stage IV SUVR	−0.56 (−0.65 to −0.47)	<0.001	−0.60 (−0.68 to −0.51)	<0.001
Braak Stage V SUVR	−0.5 (−0.60 to −0.39)	<0.001	−0.55 (−0.63 to −0.44)	<0.001
Braak Stage VI SUVR	−0.47 (−0.57 to −0.36)	<0.001	−0.49 (−0.59 to −0.38)	<0.001

a *P*-values are for Pearson correlation coefficients. Two-sided 95% confident intervals are shown in brackets.

SUVR, standardized uptake value ratio; DR, delayed recall; d′, discriminability (recognition).

In addition, we hypothesized that PET-Braak stage SUVR in early stages would be related to delayed recall memory scores and PET-Braak SUVR in late stages would be coupled with recognition memory scores. Linear regression analyses showed that the combination of delayed recall and recognition scores as well as covariates significantly predicted tau PET SUVR in all PET-Braak stages (Tables 3–5). It was revealed that, within models, RAVLT delayed recall scores accounted for a significant amount of variance in PET-Braak Stage I SUVR (*β* = −0.27, *t*(203) = −3.46, *P* < 0.001), PET-Braak Stage II SUVR (*β* = −0.23, *t*(203) = −2.83, *P* = 0.005) and PET-Braak Stage III SUVR (*β* = −0.20, *t*(203) = −2.23, *P* = 0.025), while RAVLT d′ scores explained a significant amount of variance in PET-Braak Stage III SUVR [*β*=−0.24, *t*(203) = −2.77, *P* < 0.006], PET-Braak Stage IV SUVR [*β*=−0.24, *t*(203) = −2.7, *P* = 0.007] and PET-Braak Stage V SUVR [*β*=−0.23, *t*(203) = −2.48, *P* = 0.014]. The analyses accounted for the effect of amyloid-β, age, sex, years of education, *APOE* status and RAVLT C′ scores, a measurement of response bias.^48^ Full model statistics are presented in Tables 3–5.

**Table 3:** Regression coefficients of memory scores on tau SUVR of Braak Stages I and II^a^

	Braak I SUVR			Braak II SUVR		
	Beta (95% CI)	*T*-value	*P-*value	Beta (95% CI)	*T*-value	*P-*value
RAVLT delayed recall z-scores	−0.27 (−0.42 to −0.11)	−3.46	**<0**.**001**	−0.23 (−0.39 to −0.07)	−2.83	**0**.**005**
RAVLT recognition d′ scores	−0.15 (−0.30–0.01)	−1.94	0.054	−0.11 (−0.27 to 0.05)	−1.37	0.171
RAVLT C bias scores	−0.01 (−0.09 to 0.08)	−0.12	0.903	−0.03 (−0.05 to 0.12)	0.8	0.427
Neocortical [^18^F]AZD4694 SUVR	0.45 (0.34 to 0.55)	8.43	<0.001	0.48 (0.37 to 0.59)	8.64	<0.001
Sex (male)	−0.23 (−0.40 to −0.05)	−2.59	0.01	−0.22 (−0.41 to −0.04)	−2.4	0.017
Age	−0.09 (−0.18 to −0.01)	−2.22	0.028	−0.10 (−0.18 to 0.01)	−2.14	0.034
Years of education	0.02 (−0.06 to 0.11)	0.6	0.549	0.04 (−0.04 to 0.13)	1.01	0.313
APOE ε4	0.30 (0.12 to 0.47)	3.30	0.001	0.3 (0.11 to 0.49)	3.18	0.002

a Adjusted *R*^2^: 0.67, F stat = 53.6 (Braak I); Adjusted *R*^2^: 0.63, F stat = 44.9 (Braak II). *P*-values in bold indicate statistical significance for memory scores.

**Table 4:** Regression coefficients of memory scores on tau SUVR of Braak Stages III and IV^a^

	Braak III SUVR			Braak IV SUVR		
	Beta (95% CI)	*T*-value	*P-*value	Beta (95% CI)	*T*-value	*P-*value
RAVLT delayed recall z-scores	−0.20 (−0.38 to −0.03)	−2.26	**0**.**025**	−0.15 (−0.33 to 0.03)	−1.67	0.097
RAVLT recognition d′ scores	−0.24 (−0.42 to −0.07)	−2.77	**0**.**006**	−0.24 (−0.41 to −0.06)	−2.7	**0**.**007**
RAVLT C bias scores	0.09 (−0.01 to 0.18)	−1.89	0.06	−0.05 (−0.14 to 0.05)	−0.98	0.326
Neocortical [^18^F]AZD4694 SUVR	0.32 (0.20 to 0.44)	5.27	<0.001	0.35 (0.23 to 0.47)	5.68	<0.001
Sex (male)	−0.16 (−0.37 to 0.04)	−1.62	0.108	−0.2 (−0.4 to 0.01)	−1.95	0.053
Age	−0.30 (−0.39 to −0.20)	−6.05	<0.001	−0.33 (−0.42 to −0.23)	−6.59	<0.001
Years of education	0.07 (−0.02 to 0.17)	1.50	0.135	0.06 (−0.04 to 0.15)	1.14	0.216
APOE ε4	0.04 (−0.17 to 0.24)	0.35	0.726	−0.02 (−0.23 to 0.19)	−0.17	0.863

a Adjusted *R*^2^: 0.55, F stat = 33.5 (Braak III); Adjusted *R*^2^: 0.54, F stat = 32.1 (Braak IV). *P*-values in bold indicate statistical significance for memory scores.

**Table 5:** Regression coefficients of memory scores on tau SUVR of Braak Stages V and VI^a^

	Braak V SUVR			Braak VI SUVR		
	Beta (95% CI)	*T*-value	*P-*value	Beta (95% CI)	*T*-value	*P-*value
RAVLT delayed recall z-scores	−0.12 (−0.31 to 0.07)	−1.27	0.206	−0.16 (−0.36 to 0.04)	−1.55	0.123
RAVLT recognition d′ scores	−0.23 (−0.42 to −0.05)	−2.48	**0**.**014**	−0.17 (−0.37 to 0.03)	−1.72	0.088
RAVLT C bias scores	−0.04 (−0.14 to 0.06)	−0.78	0.435	0.09 (−0.02 to 0.20)	1.64	0.103
Neocortical [^18^F]AZD4694 SUVR	0.31 (0.19–0.44)	4.8	<0.001	0.33 (0.20 to 0.47)	4.76	<0.001
Sex (male)	−0.21 (−0.43 to 0.01)	−1.94	0.053	−0.15 (−0.38 to 0.08)	−1.26	0.208
Age	−0.38 (−0.49 to −0.28)	−7.27	<0.001	−0.32 (−0.43 to −0.21)	−5.64	<0.001
Years of education	0.09 (−0.01 to 0.19)	1.74	0.083	0.1 (−0.01 to 0.21)	1.74	0.084
APOE ε4	−0.08 (−0.30 to 0.13)	−0.76	0.448	−0.20 (−0.43 to 0.04)	−1.64	0.102

a Adjusted *R*^2^: 0.49, F stat = 25.8 (Braak V), Adjusted *R*^2^: 0.41, F stat = 19.1 (Braak VI). *P*-values in bold indicate statistical significance for memory scores.

**Figure 3:**
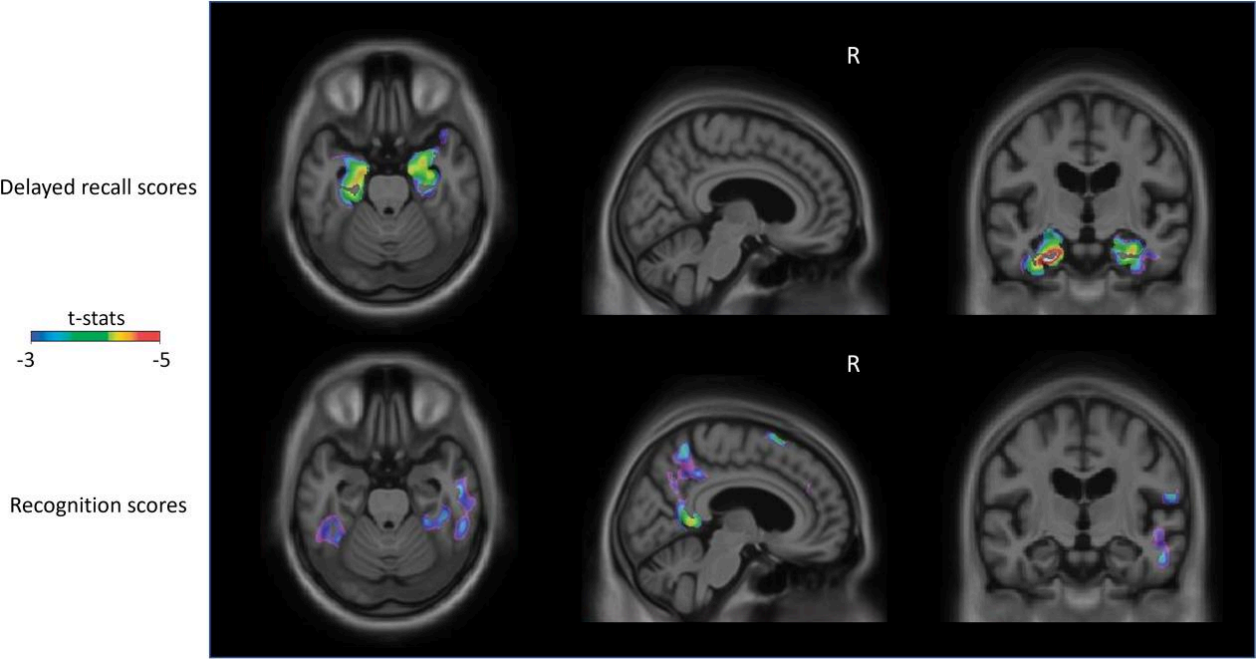
**PET scans showing that delayed recall scores are associated with tau deposition in antero-mesial regions, while recognition scores relate with tau in posterior temporal and medial parietal cortices when correcting for the remaining memory scores.** T-statistical parametric maps were corrected for multiple comparisons using a random field theory cluster threshold of *P* < 0.001, overlaid on the Alzheimer'’ disease neuroimaging initiative reference template. Two linear regression models were used, where either delayed recall z-scores or recognition d′ scores were entered as outcome variables and [^18^F]-MK6240 as predictor. RAVLT response bias C′ scores, age, sex, years of education, *APOE* status, amyloid-β positivity and either delayed recall z-scores or recognition d′ scores were used as covariates.

Finally, to further explore the idea that delayed recall memory is more associated with tau in areas pertaining to early PET-Braak stages, while recognition memory relates to tau in late PET-Braak stage areas, we conducted yet another set of voxel-wise analyses with either delayed recall or recognition scores as outcome variables and [^18^F]-MK6240 as predictor. We corrected for amyloid-β positivity, age, sex, years of education, *APOE* status and C′ scores. We found that delayed recall scores significantly correlated with the accumulation of tau in anterior MTL regions including the hippocampal complex that largely correspond to PET-Braak I, II and III, while recognition scores significantly related to tau at posterior cortical areas falling mainly in PET-Braak III, IV and V, but also some PET-Braak VI regions (Fig. 3; random field theory corrected at *P* < 0.001).

## Discussion

We applied a recent PET-Braak framework approach to evaluate stage-specific deficits in verbal memory formation across Alzheimer’s disease. We showed that delayed recall and recognition memory impairment characterize PET-Braak Stage IV. Brain areas where tau appears to differentially affect the creation of verbal memories are temporal, medial parietal, temporoparietal and prefrontal cortex. While delayed recall performance was associated with tau load in anterior medial temporal areas, recognition memory capacity related to tau pathology predominantly in posterior neocortical regions. While the early spread of tau pathology may be associated with minor but detectable changes in verbal memory function reported in aging and prodromal Alzheimer’s disease populations, tau spread in PET-Braak Stages III–IV and beyond may contribute to the striking drop in verbal memory performance observed in clinical and some preclinical Alzheimer’s disease patients.^33^

Our results showed that a substantial memory deficit arises between PET-Braak III and PET-Braak IV, and that delayed recall alone may be affected in some individuals at earlier stages. The former findings are in line with studies in which a large memory decline is seen in patients with tau pathology in composite regions corresponding to PET-Braak III–IV *in vivo*.^6,7,9,21,54^ The presence of subtle verbal memory decline in early PET-Braak stage participants also matches abundant literature on delayed recall weakening as an early cognitive sign of unfolding Alzheimer’s disease before clinical diagnosis is established.^10,55-66^ More recent work specifically found that subtle verbal memory impairment is seen in individuals assigned to PET-Braak II *in vivo*^6^ and is associated with tau load in areas classically considered Braak I and II.^21^ A few studies have previously indicated that only delayed free recall is compromised in healthy aging (for a review, see Koen and Yonelinas),^33^ and that further delayed recall dysfunction is observed prior to familiarity-based recognition decay in disease.^67-69^ Nonetheless, these findings are in contrast with other research that shows both types of memory simultaneously decline in MCI and Alzheimer’s disease populations.^70-72^ We propose that using PET-Braak staging rather than categorical diagnosis may more accurately assist in defining the progression of memory loss across preclinical and symptomatic phases of Alzheimer’s disease.

When exploring the regions of the brain where the accumulation of tau impacts memory formation, we observed that tau uptake (SUVR) displayed the strongest associations with delayed recall in PET-Braak Stage I, gradually decreasing correlation across higher PET-Braak stages. As for recognition, correlations with tau peaked at PET-Braak Stage I, were similarly strong for PET-Braak II through IV, and remained above *P* = 0.5 at PET-Braak V. Despite puzzling results for the significance of differences between correlations, these exploratory analyses suggest that tau load in early PET-Braak stages might be most relevant to delayed recall, and that tau spread through late PET-Braak regions consistently impact recognition memory.

Our unbiased voxel-wise analyses generally validated this conclusion. Delayed recall and recognition memory were affected by tau pathology in virtually identical regions including MTL, anterior and lateral temporal cortex, posterior and medial parietal cortices and prefrontal cortex. Bearing in mind that delayed recall and recognition are conceptually and practically intermingled, this finding is not surprising. Previous work has reported that both involve activity in the same brain networks in CU elderly and MCI patients.^15,74^ Nevertheless, we found that the strength of the association between tau and memory scores across the brain seems to depend on the nature of memory. This may not be surprising either since, albeit related, delayed recall and recognition memory implicate different degrees of effort and resources.^15,74,75^ Several studies have indicated that tau pathology in the MTL is associated with deficits in delayed recall performance,^76^ but there is also some research proving that tau pathology or its impact beyond these areas correlates with memory performance in MCI and/or Alzheimer’s disease patients.^21,77,78^ Moreover, recent literature has shown that tau pathology is also linked to recognition memory underperformance,^79^ but no tau PET research has examined recognition in detail to the best of our knowledge. Functional imaging studies have highlighted the importance of areas such as the prefrontal cortex, posterior parietal cortices, occipitotemporal areas and fusiform gyrus for recognition besides MTL structures in experiments using recognition memory paradigms.^80-85^ Our findings thus seem to be consistent with the notion that the spread of tau along the Alzheimer’s disease spectrum has an impact on broader memory systems that encompass MTL, prefrontal cortex, lateral temporal and posterior neocortical regions.^75^ Most importantly, distinct aspects of verbal memory are differentially impeded by tau aggregation in these brain areas.

We further hypothesized that anterior MTL tauopathy enables delayed recall memory failure, while posterior-medial tauopathy hinders recognition memory. To do so, we first ran regression analyses by PET-Braak region SUVR, where we controlled for the effect of the remaining memory type (i.e. we included both memory scores in our models) as well as potentially relevant variables. And, secondly, we run another set of voxel-wise analyses accounting for the effect of the remaining memory type as well. Regression analyses confirmed that PET-Braak I–III SUVRs were tied to delayed recall, and that PET-Braak III-V SUVRs were linked to recognition. In line with regression analyses, voxel-wise analyses showed that regions of the anterior MTL, including entorhinal and transentorhinal cortex, as well as hippocampus, displayed the strongest associations with delayed recall, whereas tau PET uptake in lateral temporal and posterior-medial systems did so for recognition. Taken together, our results corroborated the concept that tau buildup in early PET-Braak stages is coupled with delayed recall deficits, while tau in PET-Braak Stage III and beyond is accompanied by recognition shortfalls. Importantly, we found no associations between amyloid PET and memory scores across the brain, which indicates deficits were independent of the effects of amyloid pathology. Literature has previously suggested that cognitive decline is associated with tau accumulation, rather than amyloid.^86-88^

The debate on whether delayed recall and recognition rely on the same brain areas and decline in parallel in Alzheimer’s disease is complex and controversial.^33^ Besides the fact that divergent findings may be due to methodological, sample or measurement-related differences,^33,89^ in literature, a distinction is made between memory based on recollection and memory based on familiarity.^33,68,72,90-95^ Delayed recall, as well as some forms of recognition, would heavily rely on recollection.^75^ Familiarity-based memory would be a shallower type of process that is concerned in item recognition tests. Our results on recognition seem to strongly implicate areas classically considered to mediate recollection. Some literature indicates that Yes/No recognition tests, such as RAVLT’s, supposedly tap on recollection.^90^ Since an important body of research has shown that recollection is corrupted in healthy aging as well as preclinical and clinical Alzheimer’s disease,^33,97^ it was reasonable to expect that fluctuation of scores for the RAVLT recognition test would go hand-in-hand with variation in delayed recall scores, also seemingly dependent on recollection. However, our goal was not to compare young adults against healthy elderly, nor did we define our groups based on clinical diagnosis. Instead, we compared participants using the biologically defined PET-Braak continuum,^6,39^ where elderly with tau accumulation below a predetermined cutoff in PET-Braak I^36,39^ was the reference group (PET-Braak 0). It is in this context that we found significant decrease of delayed recall and not recognition in early PET-Braak stages. Studies employing diagnostic categories may put together patients with underlying tau pathology of various degrees, and it is well-known that CU elderly may be affected by tau pathology (as mentioned, most of our PET-Braak I and PET-Braak II participants are CU; but see also Harrison *et al.*,^98^ Jack *et al.*,^99^ Josephs *et al.*,^87^ Maass *et al.*^20^ and Marks *et al.*^100^). When we used young participants as the reference group, we found a significant decline in performance for both tests in PET-Braak 0 participants (Supplementary Fig. 3). When using the same reference group and diagnostic categories instead of PET-Braak staging, we did not detect differences between delayed recall and recognition (Supplementary Fig. 4). Neither approach would have helped us elucidate the subtle differences in performance that we found. Staging that relies on *in vivo* tau PET to segregate participants may enable us to make finer judgements on the evolution of decay in verbal memory construction along Alzheimer’s disease.

Our findings are consistent with existing memory systems’ models. In their review, Ranganath and Ritchey^75^ make a clear distinction between an anterior-temporal (AT) system, that would mediate familiarity-based recognition responses, and a posterior-medial (PM) system, that would deal with recollection-based recognition responses. The AT system incorporates the PRC, the lateral orbitofrontal cortex, the ventral temporopolar cortex and the amygdala. The PRC is central in this system. Importantly, the PRC contains the transentorhinal cortex,^100^ which is one of the regions first to be affected by Alzheimer’s disease-related tauopathy.^45^ The PM system comprises the PHC and the retrosplenial cortex as core components, and anterior thalamus, mamillary bodies, pre- and parasubiculum, posterior cingulate cortex, precuneus, lateral parietal and medial PFC as additional elements. Since our PET imaging results showed that tau aggregation in early PET-Braak stages, which include transentorhinal cortex, is less associated with recognition scores than tau burden in later-stage areas that include posterior cingulate cortex/retrosplenial cortex/precuneus, parietal cortex and PHC, our findings support the assumption that the recognition portion of RAVLT is more dependent on recollection processes that rely on the PM system.^28,73,75,82,83,94,102,103^ Except for areas where [^18^F]-MK6240 does not show a robust signal for neurofibrillary tangles (e.g. thalamus),^36,39^ recognition memory showed some of its strongest associations with tau PET in posterior cingulate cortex/retrosplenial cortex/precuneus, lateral parietal and PHC regions of the PM system. Since PET-Braak I includes the PRC, which is an essential part of the AT system, the strength of the correlation between PET-Braak I SUVR and recognition scores (Table 2), together with a lack of recognition memory impairment in people assigned to PET-Braak Stages I, II and III (Fig. 1), may be indicative of a switch from recollection-based responses to familiarity-based responses in Alzheimer’s disease that has been previously reported.^72^

The hippocampus, which operates as a link between AT and PM systems, is believed to play a role in both encoding and retrieval processes.^27,28,73,104-107^ Based on our outcomes, it seems to have a less prominent role than posterior neocortical areas in recognition memory (Fig. 2). Yet, it appears that the hippocampus is critical to delayed recall. Literature has not reached unequivocal consensus on whether Alzheimer’s disease memory deficits are due to encoding or retrieval.^97,108-110^ While we cannot unravel memory processes at play based on tau PET retention since it is not functionally associated with performance, it may be speculated that, at least at early stages where only delayed recall is altered, Alzheimer’s disease tauopathy is primarily threatening to retrieval processes.

Congruent with the idea that the hippocampus has a more prominent job integrating information from and relaying it to different brain areas, we propose that early Alzheimer’s disease-related tauopathy in hippocampus and surrounding structures is enough to obstruct delayed free recall but, when some contextual information is provided, the hippocampus has an extra support that enables it to perform well enough for recognition to be spared. It is only when structures of the PM system are reached by tauopathy (i.e. PET-Braak Stages III–IV) that recognition, assumed to primarily rely on recollection, follows delayed recall’s fate. At this point, two explanations are possible. Perhaps damage in the PM system alone explains recognition memory decay. Alternatively, further tau accumulation in anterior MTL, followed by neurodegeneration, strips it off its mnemonic functions. When this occurs, patients are no longer able to store information (i.e. encoding processes are hindered), and it is then that verbal memory as a whole dramatically drops.

The external validity of our findings is biased by a number of methodological limitations. First, while we imply that our work provides an insight to the evolution of memory decline along the Alzheimer’s disease spectrum, we have not conducted longitudinal analyses. Secondly, the number of participants in some PET-Braak stages was low, which may have affected the robustness of our results on group comparisons. Moreover, although a significant amount of studies has suggested that verbal memory is the first or most prominent cognitive indicator of incipient Alzheimer’s disease, exploring whether other cognitive domains may be affected using PET-Braak staging *in vivo* may be enlightening.^3,111^ Furthermore, we have focused on a single, though robust, memory test. It would be interesting to replicate these results on similar verbal memory tests (e.g. California Verbal Learning Test) or modalities, since we have only examined verbal memory. Also, setting the scope of our investigation on tau pathology may have prevented us from identifying brain areas that seem to be potentially vital to memory^112^ but that do not exhibit extensive [^18^F]-MK6240 binding. Finally, the significance of differences between participants at PET-Braak Stage 0 and participants at PET-Braak Stages I–II should also be cautiously observed, given its low magnitude.^113^ Indeed, where results were not significant, there seems to be a trend for recognition scores (adjusted *P* = 0.066 for PET-Braak 0 versus PET-Braak II). A possible source of bias in our results may be the manner in which recognition performance was measured. Nevertheless, we replicated differences when using the two-high threshold model^48^ to calculate recognition scores as well (Supplementary Fig. 2). There is also the possibility that RAVLT memory tests produce a ceiling effect that does not allow to examine verbal memory to the full extent,^114,115^ although this possibility seems unlikely in our study, since analyses including young participants showed that average recognition scores could be significantly higher than the ones from PET-Braak 0 participants (Supplementary Fig. 3).

Our study left a few unanswered questions. First, longitudinal studies should be conducted to see if our results are replicated. Secondly, distinctive aspects of memory should be handled separately to allow for a detailed understanding on how memory relates to underlying brain structures affected by tau. Prior tau PET studies have frequently used composite memory scores.^6,7,9,20,76^ While this approach may be fruitful to portrait a general picture of the relationship between tau and memory, a more detailed understanding entails specialized attention to distinct aspects of this cognitive domain (an example would be the work of Digma *et al.*^78^). It is patent that lack of consensus across verbal memory manuscripts may be related to the diversity of methods used,^72,89^ but it is also true that this diversity may help to examine memory from different perspectives.

In summary, our findings provide evidence that verbal memory dysfunction in Alzheimer’s disease populations may begin with a barely noticeable change in delayed recall due to tau accumulation. As the disease advances, concomitant with the spread of tau beyond entorhinal, transentorhinal and hippocampus allocortices, verbal memory suffers a major blow that compromises its whole integrity. *In vivo* PET-Braak staging may be more accurate than a clinical diagnosis in determining when and how different aspects of verbal memory are affected along the Alzheimer’s disease spectrum, which may be of assistance to differential diagnosis efforts, as well as to decisions on treatment options and clinical trials’ recruitment.

## Supplementary Material

fcad146_Supplementary_Data

## Data Availability

The data presented in this study are available from the corresponding author upon reasonable request, and such arrangements are subject to standard data-sharing agreements.

## Abbreviations

AT =: anterior temporal
C =: recognition bias scores
CU =: cognitively unimpaired
d′ =: recognition discriminability scores
mCi =: millicuries
MCI =: mild cognitive impairment
MTL =: medial temporal lobe
PFC =: prefrontal cortex
PHC =: parahippocampal cortex
PM =: posterior medial
PRC =: perirhinal cortex
RAVLT =: Rey auditory verbal learning test
SD =: standard deviation
SUVR =: standardized uptake value ratio
