# Supplementary material for: Verbal memory formation across PET-based Braak stages of tau accumulation in Alzheimer’s disease
Source: Brain Commun. 2023 May 18;5(3):fcad146. doi: 10.1093/braincomms/fcad146 (PMC10213301; doi:10.1093/braincomms/fcad146)
Supplement: fcad146_Supplementary_Data [file fcad146_supplementary_data.docx]

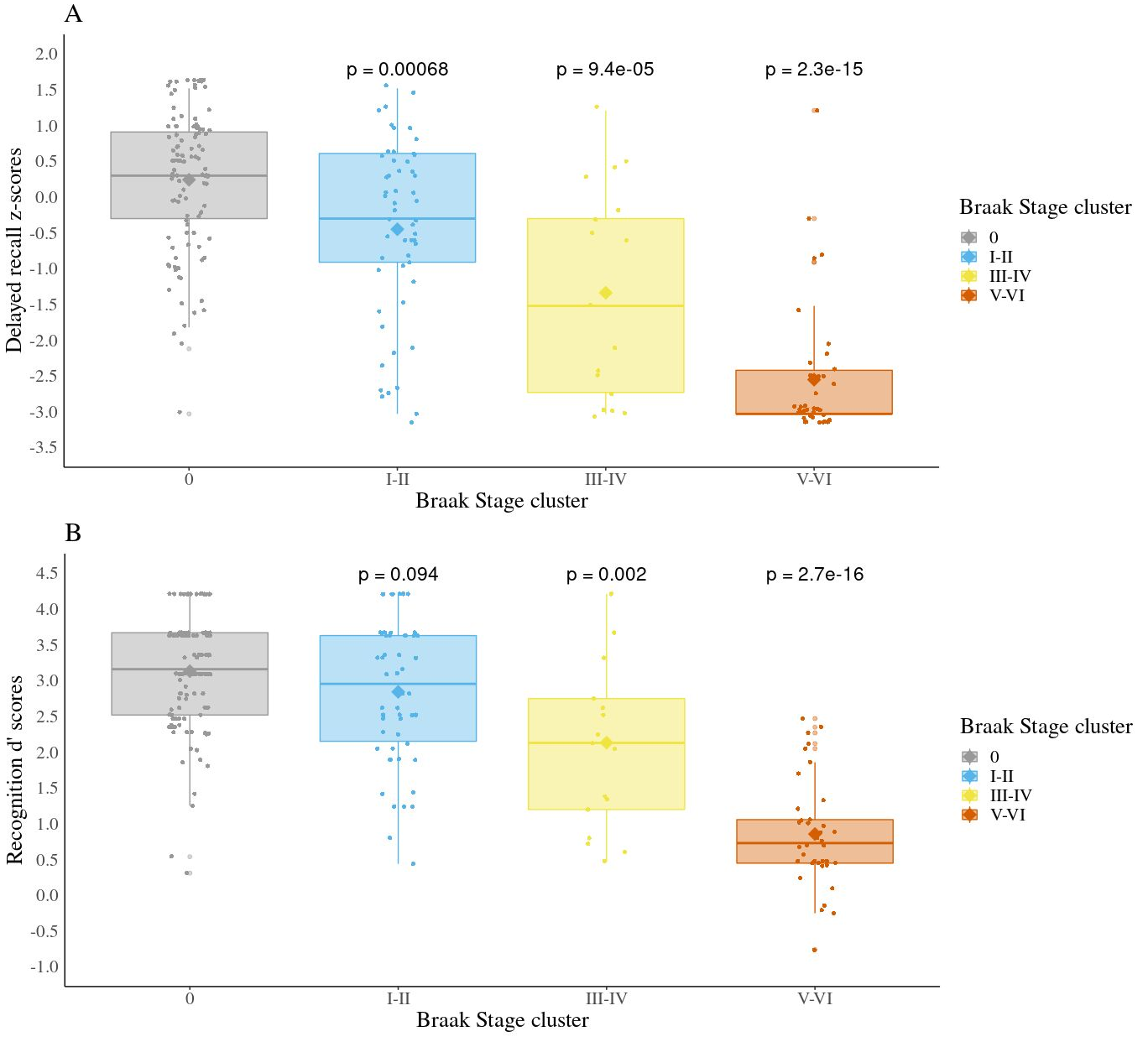


**Supplementary Figure 1 Delayed recall declines at PET-Braak stage cluster I-II, and recognition declines at PET-Braak stage cluster III-IV. Mean and median memory scores as assessed by in vivo tau PET. (A)** RAVLT delayed recall z-scores. **(B)** RAVLT d’ scores. Adjusted p-values are displayed for the comparison between individuals in Braak 0 and individuals in more advanced Braak stages. Kruskal-Wallis H tests were applied. For multiple comparisons, we used Mann-Whitney U tests, with FDR correction. 0: cognitively unimpaired elderly with no significant tau; I-II, III-IV, V-VI: PET-Braak stage cluster. Scores from cognitively unimpaired elderly were used to calculate z-scores.


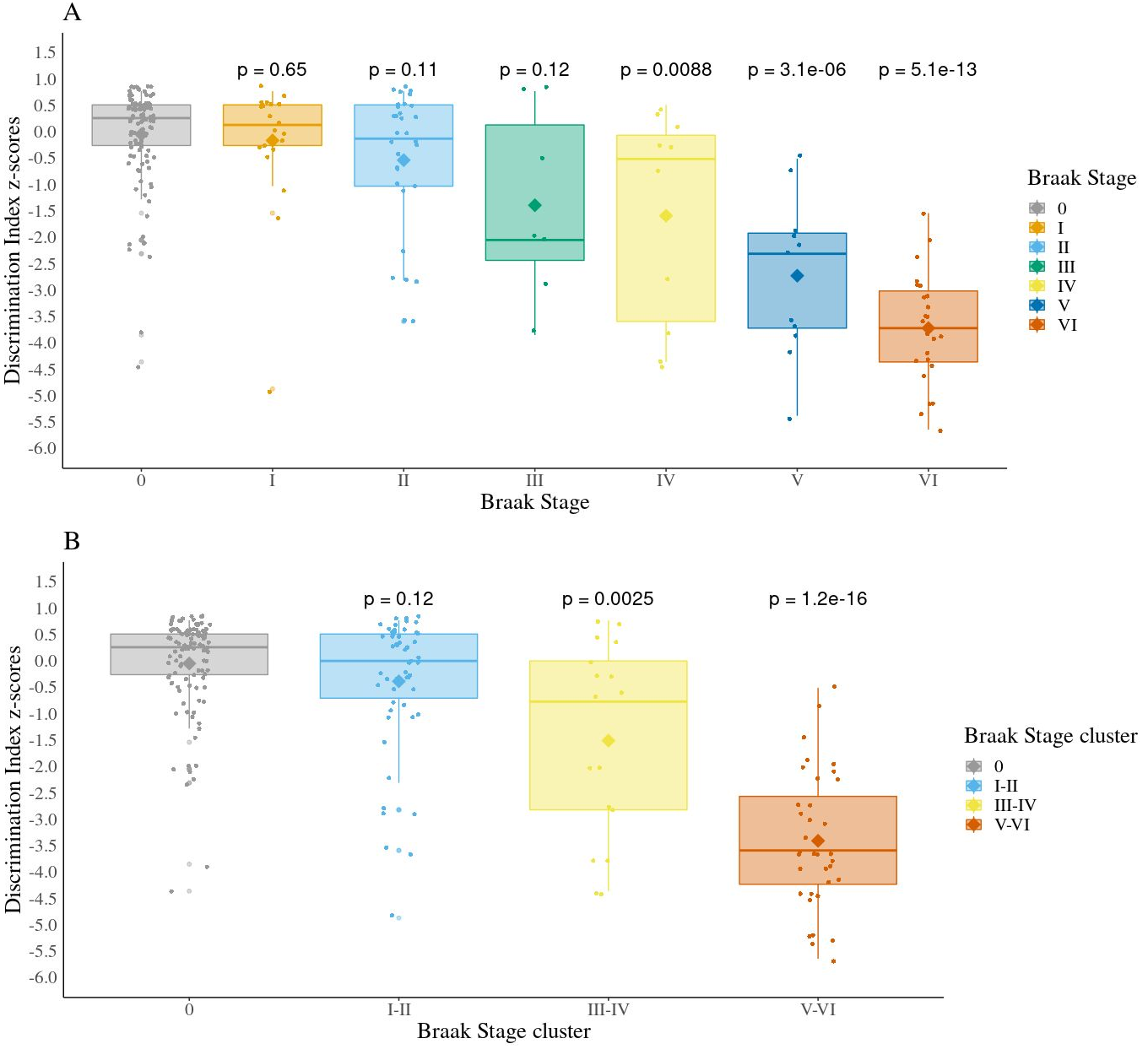


**Supplementary Figure 2 Mean and median recognition scores by Braak stage as assessed by in-vivo tau PET using the two-high threshold model. (A)** RAVLT-derived Discrimination Index z-score by Braak stage. **(B)** RAVLT-derived Discrimination Index z-score by Braak stage cluster. Adjusted p-values are displayed for the comparison between individuals in Braak 0 and individuals in more advanced Braak stages. Kruskal-Wallis H tests were applied. For multiple comparisons, we used Mann-Whitney U tests, with FDR correction. 0: cognitively unimpaired elderly with no significant tau; I-VI: PET-Braak stage; I-II, III-IV, V-VI: PET-Braak stage cluster. Scores from cognitively unimpaired elderly were used to calculate z-scores.


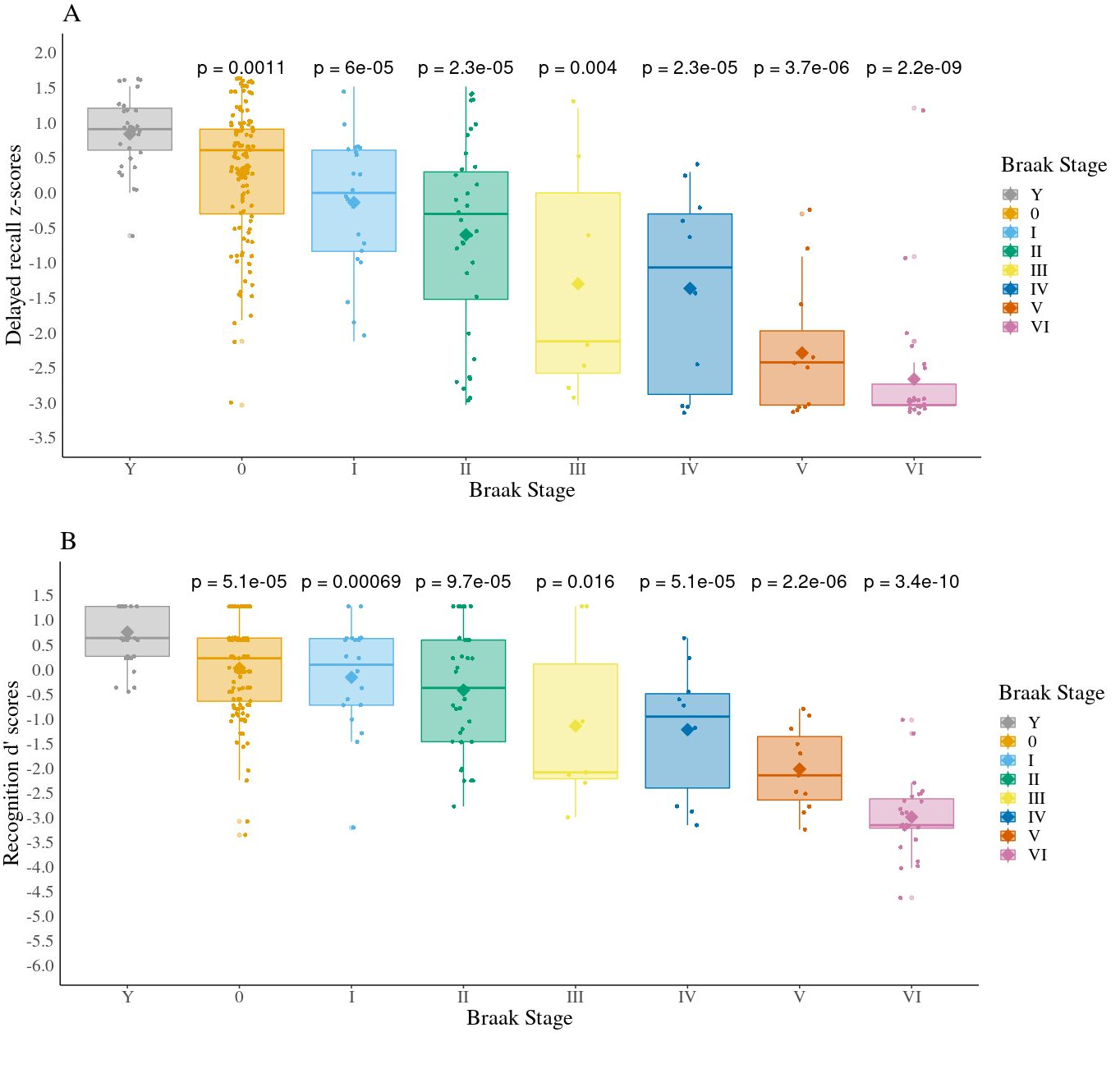


**Supplementary figure 3 Mean and median memory scores by Braak stage as assessed by in vivo tau PET with young participants as the control group. (A)** RAVLT delayed recall z-scores. **(B)** RAVLT d’ z-scores. Adjusted p-values are displayed for the comparison between young individuals and elderly individuals without (Braak 0) and with tau (Braak stages). Kruskal-Wallis H tests were applied. For multiple comparisons, we used Mann-Whitney U tests, with FDR correction. 0: cognitively unimpaired elderly with no significant tau; Y: young participants; I-VI: PET-Braak stage. Scores from cognitively unimpaired elderly were used to calculate z-scores.


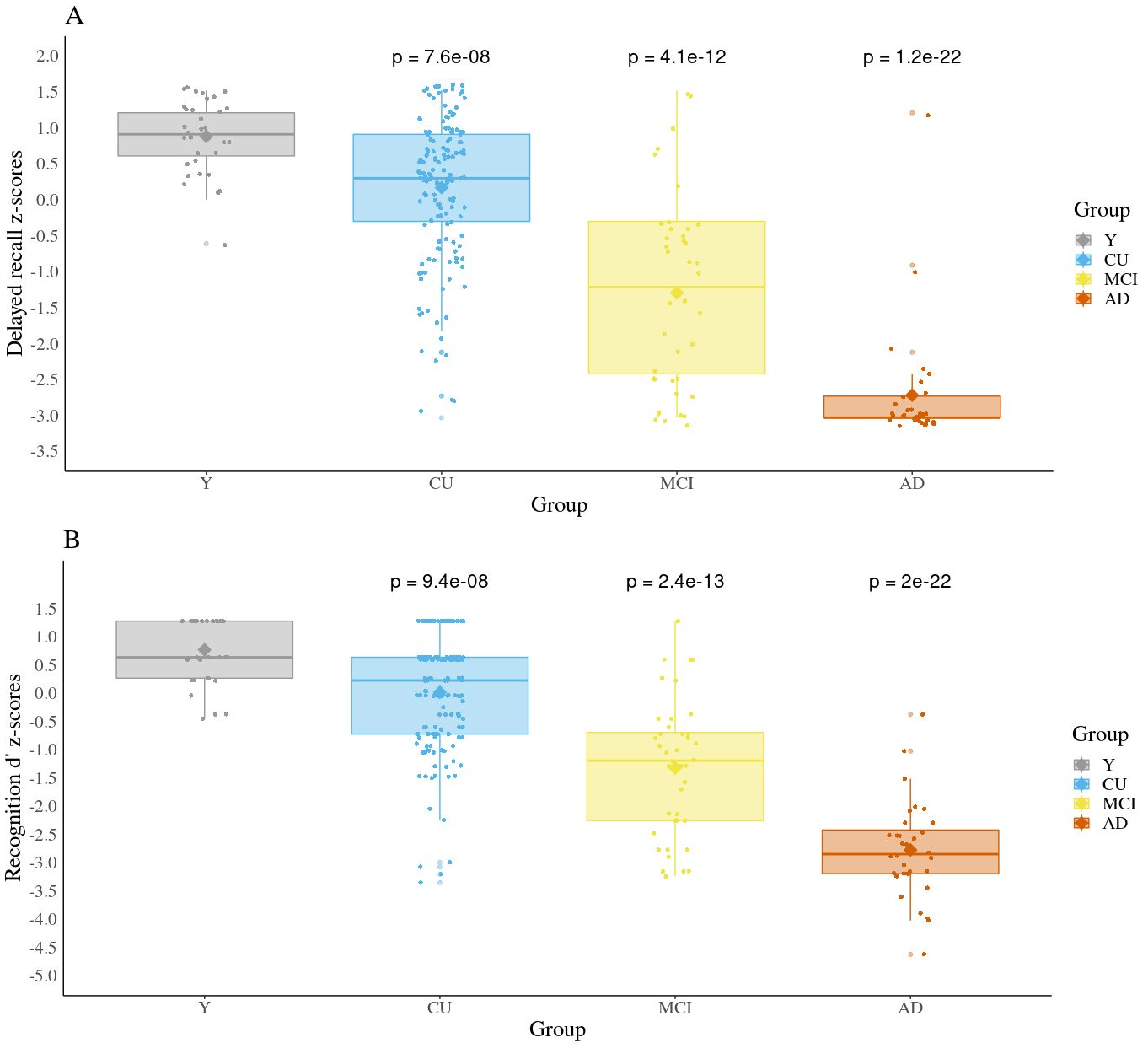


**Supplementary figure 4 Mean and median memory scores by diagnosis as assessed by in vivo tau PET with young participants as the control group. (A)** RAVLT delayed recall z-scores. **(B)** RAVLT d’ z-scores. Adjusted p-values are displayed for the comparison between young individuals and elderly individuals without (Braak 0) and with tau (Braak stages). An analysis of variance (ANOVA) was conducted. T-test planned contrasts with FDR correction for multiple comparisons were applied. Y: young participants; CU: cognitively unimpaired elderly; MCI: mild cognitive impairment participants; AD: Alzheimer’s disease patients. Scores from cognitively unimpaired elderly were used to calculate z-scores.
